# One-Step Surface Immobilization of Protein A on Hydrogel Nanofibers by Core-Shell Electrospinning for Capturing Antibodies

**DOI:** 10.3390/ijms22189857

**Published:** 2021-09-12

**Authors:** Chihiro Naganuma, Kosuke Moriyama, Shin-ichiro Suye, Satoshi Fujita

**Affiliations:** 1 Department of Frontier Fiber Technology and Science, University of Fukui, Fukui 910-8507, Japan; naganumac@biofiber-fukui.com (C.N.); mori6341@gmail.com (K.M.); suyeb10@u-fukui.ac.jp (S.-i.S.); 2 Organization for Life Science Advancement Programs, University of Fukui, Fukui 910-8507, Japan

**Keywords:** surface modification, protein A, electrospinning, nanofiber, membrane chromatography

## Abstract

Nanofibers (NFs) are potential candidates as filter materials for affinity separation owing to their high liquid permeability based on their high porosity. Multiple and complex processes were conventionally performed to immobilize proteins for modifying NF surfaces. A simple method must be developed to immobilize proteins without impairing their biological activity. Herein, we succeeded in fabricating NFs with a core of cellulose acetate and a shell of hydrophilic polyvinyl alcohol immobilized with staphylococcal recombinant protein A by a one-step process based on core-shell electrospinning. A total of 12.9 mg/cm^3^ of antibody was captured in the fiber shell through high affinity with protein A immobilized in an aqueous environment of the hydrogel. The maximum adsorption site and dissociation constant evaluated by the Langmuir model were 87.8 µg and 1.37 µmol/L, respectively. The fiber sheet withstood triplicate use. Thus, our NF exhibited high potential as a material for membrane chromatography.

## 1. Introduction

Affinity chromatography is used to separate and purify target substances in a sample based on the affinity between the ligands and the target substances [1]. Biologically active substances, such as antibodies and lectins, are used as ligands, and various materials in which these biomolecules are immobilized are used as affinity carriers [2]. As such, for the initial purification of antibody drugs, protein A (PA) is widely used for the recovery of IgG as a ligand [3]. PA constitutes the cell wall of *Staphylococcus aureus* and consists of a single polypeptide chain with a molecular weight of 42 kDa. It has a binding domain with the Fc region of the IgG antibody. In addition, the bound antibody is readily desorbed under acidic conditions [4].

Various protein immobilization strategies reported, including covalent bond, physisorption, bioaffinity interaction, and their combinations, are summarized in excellent reviews [5,6,7,8]. Especially, for immobilizing PA, materials that are insoluble in water, possess reactive functional groups, and do not react with immobilized PA are used. Some covalent immobilizations were reported, including immobilization of PA on the surface of agarose gel carriers via glutaraldehyde [9], porous membranes composed of chitosan via glutaraldehyde [10], photopolymerized porous polyhydroxyethyl methacrylate by bromine cyanide [11], and polyvinyl alcohol (PVA) cryogel surfaces via ethylenediamine [12]. Other site-specific immobilization strategies could also be applicable [5,6,7,8].

Polymer gels, beads, and porous membranes have been widely used as immobilization carriers. The flow velocity and amount used depends on the flow rate and pressure resistance [13]. In addition, complicated surface modifications have hampered PA immobilization [14]. In recent years, nonwoven nanofiber (NF) membranes have been attracting attention as affinity carriers owing to their better permeability and resistance to pressure than membranes [13]. These properties are attributed to their high porosity; therefore, large volumes of samples can be processed at high flow rates. The high specific surface area may also significantly improve the adsorption efficiency of the carrier. In addition, the effect of molecular alignment may increase intensity [15,16]. The surface of regenerated cellulose NF membranes prepared by electrospinning was previously modified to immobilize ligand proteins [16]. However, a complex surface treatment consisting of multiple steps is necessary, which is resource and time-intensive. The reaction efficiency should also be improved to immobilize a large number of ligand proteins. Thus, a simple method to immobilize ligand proteins on the surface of nanofibers at high density is highly desired.

**Scheme 1:**
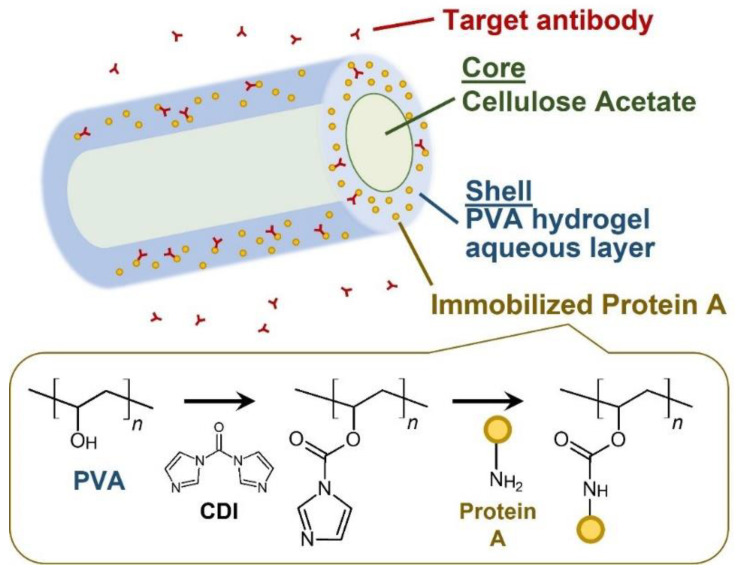
Design of the core-shell fiber with a hydrophilic hydrogel layer conjugated with PA.

In this study, we designed a novel approach, where core-shell electrospinning was used to immobilize PA on the fiber surface. Via electrospinning NFs can be fabricated by extruding a polymer solution while applying a voltage to the grounded collector [17,18]. In core-shell electrospinning, a polymer with poor spinnability can be easily fiberized by co-injecting a polymer with a good spinnability polymer through a core-shell nozzle [19,20,21]. Here, we used cellulose acetate (CA)—which has good spinnability and high mechanical strength—as the core polymer. PVA, a hydrophilic polymer with hydroxyl groups on its side chains, was used as the shell polymer. PA was covalently immobilized with PVA via hydroxyl groups. When immersed in an aqueous environment, the PVA shell hydrates to form a hydrogel layer. PA immobilized on PVA is expected to be surrounded by water and remains stable without losing the interaction between PA and the antibody. Owing to the swelling of the PVA layer, adsorption is expected to improve due to the increased contact between PA and the target antibody (Scheme 1). Conventionally, hydrogel beads such as agarose and PVA were used as the carrier. Hydrogel is suitable as a substrate for affinity chromatography because of the low non-specific adsorption of proteins [22]. However, no attempt was made to use hydrogel nanofibers because it would be difficult to obtain a tough fiber carrier consisting only of hydrogel, and nanofibers with high porosity swell in an aquatic environment, resulting in reduced porosity. This study revealed the physiological activity of the ligand as a career and the mechanical properties to withstand repeated use. This novel approach is essentially versatile and applicable to any other immobilization technique that can be used for PVA. We evaluated the antibody-binding capacity of PA-immobilized NFs as affinity carriers.

## 2. Experimental Methods

### 2.1. Fabrication of the NF Sheet

A core solution, 25% CA (Mw, 38,000; Sigma-Aldrich, Tokyo, Japan) dissolved in dimethylacetamide (DMAc) and acetone (2:1), was prepared. PVA (Mw, 146,000–186,000, Sigma-Aldrich) was added to dimethyl sulfoxide to completely dissolve while heating to 150 °C on a hot stirrer to obtain a 15% PVA solution. Then, 1,1’-Carbonyldiimidazole (CDI; Tokyo Chemical Industry, Tokyo, Japan) was added at a final concentration of 0.05%, and the mixture was stirred uniformly. Purified staphylococcal recombinant PA (30 kDa, Kaneka Corp., Tokyo, Japan) was added at a final concentration of 0.1% as a shell solution. Each polymer solution was immediately electrospun with a coaxial nozzle (NANON, MECC, Fukuoka, Japan) under an applied voltage of 2.5 kV/cm, the core flow rate of 0.4 mL/h, the shell flow rate of 0.1 mL/h, and collector rotation speed of 900 rpm.

### 2.2. Morphological Observations

To observe the fine surface structure, samples were Pt/Pd-coated (15 mA, 6 Pa, 120 s) with an ion sputter (MSP-1S, Vacuum Device Inc., Japan) and observed with a scanning electron microscope (SEM, HITACHI S-2600HS) at an acceleration voltage of 15 kV. The samples spun on the carbon support membrane were observed using a transmission electron microscope (TEM, HITACHI H-7650) at an acceleration voltage of 100–200 kV to study the internal structure of the fibers. The adsorbed antibody was visualized by immersing the samples in a 1:100 diluted fluorescent-labeled antibody (Alexa 488-rabbit anti-mouse IgG; Alexa594-rabbit anti-mouse IgG, Abcam, UK) and/or 20 µg/mL FITC-bovine albumin (Sigma-Aldrich) for 3 h at 25 °C and observing them with a fluorescence microscope.

### 2.3. Attenuated Total Reflection Fourier-Transform Infrared Spectrometry (ATR-FTIR)

The surface chemistry was analyzed using a single-reflection attenuated total reflection Fourier-transform infrared (ATR-FTIR) spectrometer on a Nicolet 6700 system (Thermo Scientific, MA, USA) in 800–2000 cm^−1^ with a resolution of 4 cm^−1^ and an average of 32 scans using a KBr detector.

### 2.4. Enzyme-Linked Immunosorbent Assay (ELISA)

A mouse IgG ELISA Quantitation Kit (Bethyl Laboratories, TX, USA) was used according to the manufacturer’s instructions. In brief, the immobilized antibody (goat-poly, anti-mouse IgG, Bethyl Laboratories) 10 µg/mL diluted with a carbonate buffer (0.05 M, pH 9.6) was added to a 96-well ELISA plate at 100 µL/well and incubated at 25 °C for 1 h. It was then washed five times with ELISA buffer (50 mM Tris, 140 mM NaCl, 0.05% Tween20, pH 8.0) and blocked with 1% bovine serum albumin for 1 h. Next, 100 µL of the target antibody (mouse, anti-goat IgG, #105-3102, Rockland Immunochemicals, PA, USA) was diluted with Blocking One (Nacalai Tesque, Kyoto, Japan) and added after washing five times with ELISA buffer for 1 h. A concentration of 0.0273 to 3.50 µg/mL was used as the standard solution for the calibration curve. After washing five times, 100 µL of labeled antibody (goat-poly, anti-mouse IgG-HRP, Bethyl Laboratories) 10 ng/mL diluted with ELISA buffer was added for 1 h. After washing five times, the samples were developed using the ELISA POD Substrate TMB kit (Nacalai Tesque, Kyoto, Japan) at 25 °C for 30 min. Subsequently, quenching with sulfuric acid (0.18 M), the absorbance was measured at 450 nm.

### 2.5. Quantification of the Antibody Absorbed

The fiber sample to be tested was cut into 5 × 5 mm pieces (0.25 cm^2^) and weighed. Mouse anti-goat IgG antibody was used as the target antibody. The samples were immersed in 100 µL of diluted target antibody, incubated at 25 °C for 1 h, and washed three times with 200 µL of ELISA buffer. This process is referred to as adsorption. Next, 600 µL of McIlvaine buffer (pH 3.0) was added to the sample, and the entire solution was recovered, in a process referred to as elution. The solution thus recovered (600 µL) was immediately neutralized by adding 300 µL of aqueous sodium hydroxide solution (2 M, pH 13.8) for the ELISA assay.

## 3. Results

### 3.1. Morphological Observation of Protein-Immobilized Core-Shell NFs

**Figure 1:**
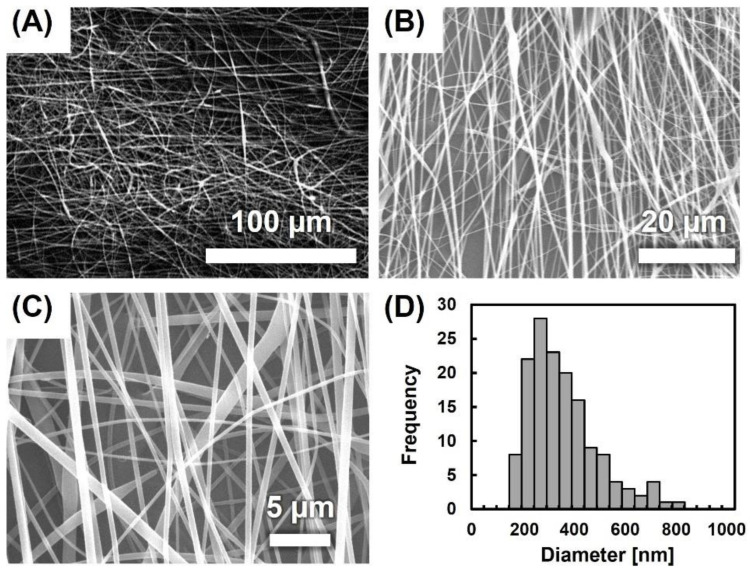
(**A**–**C**) Morphological analysis of CA/PVA + PA NF by SEM. (**D**) Distribution of the fiber diameters.

**Figure 2:**
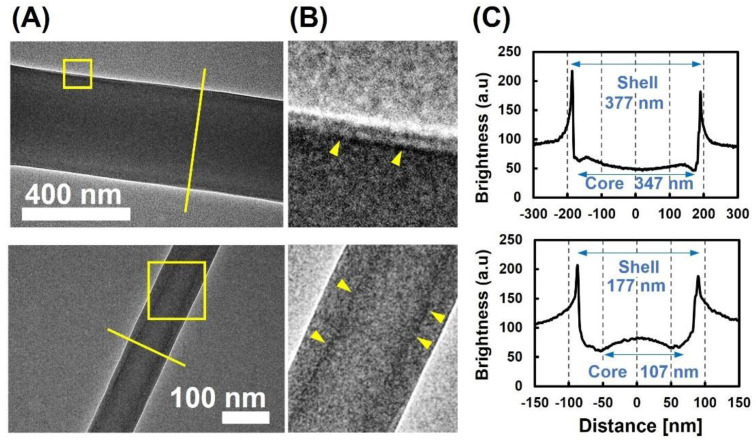
(**A**,**B**) Two representative TEM images of CA/PVA + PA NF with different diameters. Arrowheads represent the interface between the core and the shell. (**C**) The brightness distribution of the cross-section of the fiber shows the core-shell structure.

Using core-shell electrospinning, we successfully obtained a fiber with a core of CA and a shell of PVA conjugated with PA, designated as CA/PVA + PA NF. The morphology of the CA/PVA + PA NFs electrospun for 15 min was observed using SEM. Fibers with smooth surfaces and uniform diameters were obtained (Figure 1A–C). The average diameter of the fibers was 364 nm (Figure 1D). TEM was used to observe the CA/PVA + PA NF core-shell structure. The prepared CA/PVA + PA NF possessed a two-layer structure made of core and shell, which was identified by the clear interface (Figure 2A,B). The observed interface would be due to the different transmittance of the electron beam [20]. The thickness of the shell was 15–35 nm (Figure 2C). Note that it is difficult to estimate the accurate thickness of the shell from the image of a thicker fiber because a larger diameter makes the transmittance of electrons lower.

### 3.2. Fluorescent Observation of Protein-Immobilized Core-Shell NFs 

**Figure 3:**
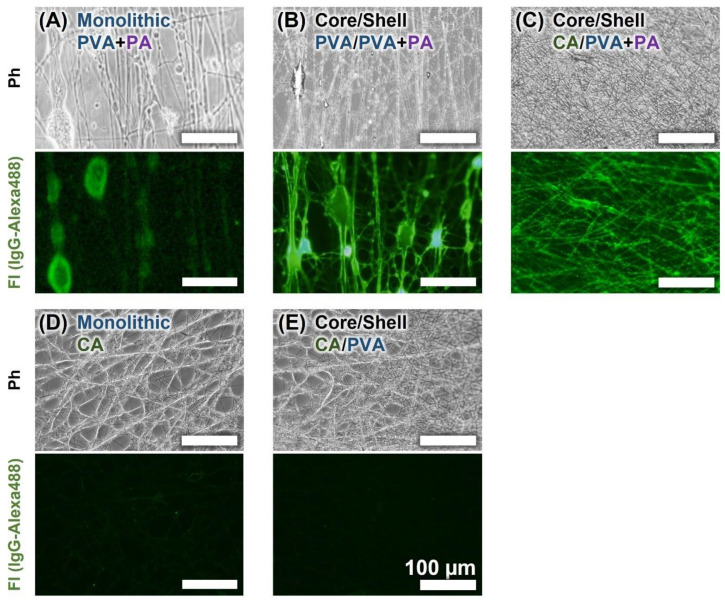
Fluorescent microscopy of fabricated NF treated with fluorescence-labeled antibody. (**A**) Monolithic PVA + PA. (**B**) Core-shell PVA/PVA + PA NF. (**C**) Core-shell CA/PVA + PA NF. (**D**) Monolithic CA. (**E**) Core-shell CA/PVA NF without the immobilization of PA.

A fluorescent-labeled antibody (Alexa Fluor 488 Rabbit Anti-Mouse IgG) was adsorbed onto the CA/PVA + PA NF and then observed under a fluorescence microscope, resulting in a uniformly stained fiber surface (Figure 3C). This demonstrates that the antibody was adsorbed onto the fiber surface, where PA was uniformly immobilized. On the other hand, the monolithic PVA + PA fiber electrospun from PVA solution containing PA and the cross-linking agent by a single nozzle had many beads and the fiber diameter was non-uniform (Figure 3A). In the fluorescent staining, the fibers swelled with water and the porous fiber structure collapsed. PVA/PVA + PA fibers electrospun with a PVA solution as the core and a PVA solution containing PA and a cross-linking agent as the shell was non-uniform in diameter (Figure 3B). Fluorescence microscopy revealed antibody binding on thick fibers, not thin fibers. This indicates that the core-shell electrospinning of PVA was not sufficiently successful. In addition, the fiber also swelled in the solution and lost its porosity.

**Figure 4:**
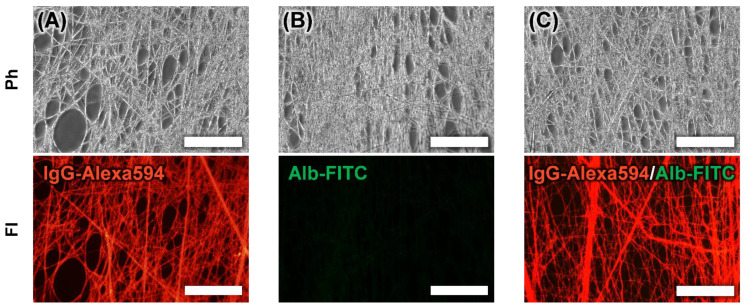
Fluorescent microscopy of core-shell CA/PVA + PA NF treated with (**A**) IgG-Alexa594 only (red), (**B**) albumin-FITC only (green), and (**C**) both of IgG-Alexa594 and Albumin-FITC (merged). Bar = 100 μm. Note that it was confirmed by preliminary experiments that the fluorescence intensity of the labeled albumin is equivalent to that of the labeled antibodies.

To investigate the effect of the non-specific adsorption, the adsorption of IgG on the core-shell CA/PVA NF without the immobilization of PA was evaluated. For comparison, the same evaluation was also performed for the CA monolithic NF. As a result, non-specific IgG adsorption was barely observed in both of them (Figure 3D,E). As a result of a similar experiment performed using fluorescently labeled albumin, no adsorption was observed (Figure 4A,B). Even when the albumin and antibody coexisted, only the antibody was selectively adsorbed but no albumin was adsorbed (Figure 4C). It is known that PVA hydrogel shows low adsorption of proteins, which was explained with the hydration layer theory [23,24]. The reason why non-specific adsorption of IgG onto the CA/PVA NF hardly occurred would be due to the blocking by the hydrated layer of PVA hydrogel on the fiber surface. From the above results, the superiority of CA/PVA + PA was shown.

### 3.3. ATR-FTIR

**Figure 5:**
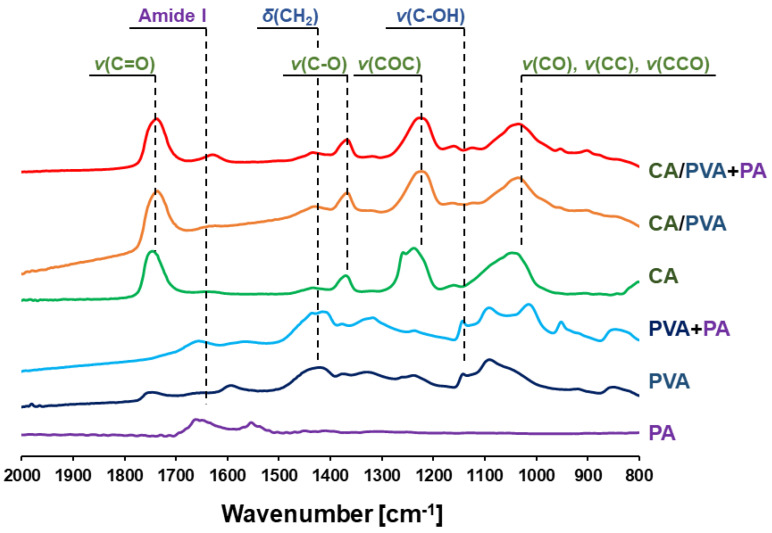
ATR-FTIR spectra of the electrospun fibers.

The fabricated CA/PVA + PA NFs were studied using a single-reflection ATR-FTIR to investigate the immobilization of PA. PVA single-layered fiber (PVA NF), CA single-layered fiber (CA NF), PA-immobilized PVA single-layered fiber (PVA + PA NF), and CA/PVA core-shell fiber without PA (CA/PVA NF) were measured as controls (Figure 5). Single-layered fibers were electrospun using a normal nozzle. PVA + PA NF were electrospun from PVA solution with PA and crosslinker—1,1’-carbonyldiimidazole—similar to CA/PVA + PA NF. Comparing the spectra of PVA NF and PVA + PA NF, the peak of Amide I derived from the covalently conjugated PA was observed at 1630–1670 cm^−1^ [25,26] in PVA + PA NF, indicating the immobilization of PA on PVA. Likewise, the peak of Amide I was also observed in the CA/PVA + PANF by comparing the spectra of CA/PVA NF and CA/PVA + PA NF, indicating the presence of PA on the surface of the core-shell fiber; this is because ATR-FTIR of core-shell NFs emphasizes surface-localized chemical information [19]. In addition, peaks derived from CA (*ν*(C=O), 1735 cm^−1^; *ν*(CO), 1220 cm^−1^; *ν*(CO), *ν*(CC), *ν*(CCO), 1035 cm^−1^) [27,28] were also observed in CA/PVA + PA NF owing to the by-products of fibers with exposed CA on the surface or fibers composed of CA only.

### 3.4. Adsorption of IgG to PA-Immobilized Core-Shell Fiber

**Figure 6:**
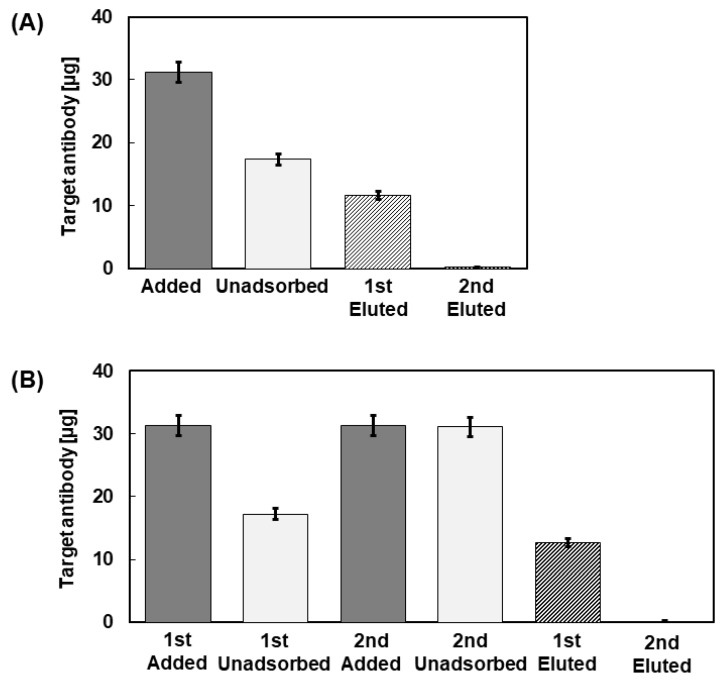
Assay of the antibody adsorbed onto CA/PVA + PA NF. The amounts of antibody adsorbed and eluted were evaluated by ELISA. (*n* = 3) (**A**) Once adsorption and following two elution processes. (**B**) Repetitive adsorption and following two elutions.

We obtained 0.22 mg of CA/PVA + PA NF sheet with an average thickness of 37 ± 3 µm by electrospinning for 90 min. The amount of loaded PA was estimated as 0.87 mg per g of NF sheet. We then cut the sheet and tested adsorption. First, the amount of the target antibody (mouse anti-goat IgG) adsorbed was quantified via sandwich ELISA. Then, 300 µg/mL of the target antibody solution was added to the fiber for adsorption, and the amount of unadsorbed target antibody was measured. After the first elution, the amount of the eluted antibody was measured. The elution was repeated and the quantity of eluted antibodies was measured. The amount of antibody added and unadsorbed, initial elution, and final elution amounts were obtained as 31.2 ± 2.1, 17.4 ± 0.5, 11.7 ± 0.2, and 0.222 ± 0.009 µg, respectively (Figure 6A). The total unadsorbed and the eluted amount was 29.3 ± 0.7 µg, which was 94% of the total amount added. This indicates that there was little non-specific adsorption to the tubes and tips used, and the adsorption and elution processes were conducted quantitatively. The total eluted amount was 11.9 ± 0.2 µg, 98% of which was eluted in one operation. The area and average thickness of the CA/PVA + PA NF were 0.25 cm^2^ and 37 ± 3 µm, respectively. Considering the sheet to be a thick and porous body, the amounts of antibody per area and volume were calculated to be 47.6 µg/cm^2^ and 12.9 mg/cm^3^.

The presence of free PAs as vacant sites explains the adsorption of only about 60% of the added antibody. Therefore, to increase the amount of adsorption onto vacant PA, repetitive adsorption was performed. Likewise, in the previous experiment, the first adsorption was performed with 300 µg/mL of the target antibody. The amount of unadsorbed target antibodies was measured. Subsequently, the second adsorption was performed by adding the same concentration of the antibody. The amount of unadsorbed antibodies was also measured. The targeting antibody added in the first adsorption and the amount of unadsorbed antibody were 31.3 ± 1.5 and 17.2 ± 0.9 µg, respectively. The added and unadsorbed amounts in the second adsorption were 31.3 ± 1.3 and 31.1 ± 0.3 µg, respectively (Figure 6B). This shows that the adsorption was completed by the first adsorption. Subsequently, elution was performed twice, and the amount of eluted antibodies was measured. The amounts of the first and second elutions were 12.7 ± 0.1 and 0.205 ± 0.008 µg, respectively (Figure 6B). The total amount of antibody in the two elutes was 12.9 ± 0.1 µg, indicating that 98% of the target antibody adsorbed on the fiber was eluted at the first elution. This quantitative recovery indicates that non-specific adsorption is ignorably low. The repeated elution operations did not reduce the amount of antibody captured, which indicates that PA was immobilized tightly and not leached.

### 3.5. Evaluation of the Maximum Adsorption on the CA/PVA + PA Core-Shell Fiber

**Figure 7:**
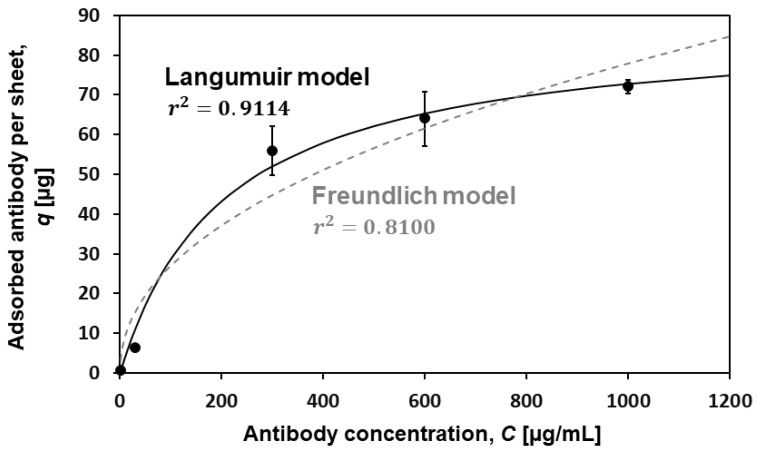
Quantitative evaluation of the antibody captured on the CA/PVA + PA NF based on the Langmuir adsorption model (black solid line) and Freundlich model (gray dotted line). (*n* = 3).

To evaluate the maximum adsorption, adsorption-elution experiments were performed at different concentrations. Figure 7 shows the relationship between the antibody concentration of the solution and the amount of eluted antibody. The adsorption of the antibody on PA was evaluated the adsorption equilibrium of the Langmuir model and Freundlich model [29,30]. The result was fitted better with the Langmuir model, where one molecule adsorbs on each adsorption site of the surface until a fully adsorbed monolayer is obtained. This is described by the following formula [31,32]:

(1) $$q=\frac{q_{max}C}{C+K}$$

where *C* is the concentration of the antibody solution added, *q* is the amount of adsorbed antibody calculated from the concentration of the eluted antibody solution, *q_max_* is the concentration of the PA adsorption site on the fiber, and *K* is the dissociation constant between PA and antibody. Fitting the experimental results to Equation (1), *q_max_*, the adsorption site of PA, and *K*, the dissociation constant, in PA-antibody adsorption were obtained. The *q_max_* of the PA adsorption site and *K* were 87.8 µg and 1.37 µmol/L, respectively. The dissociation constant obtained in this study was higher than the value reported previously (0.43 µmol/L) [33,34], possibly because the chemical immobilization of PA on the NF changes the protein structure and reduces activity.

### 3.6. Reusability as an Affinity Carrier

**Figure 8:**
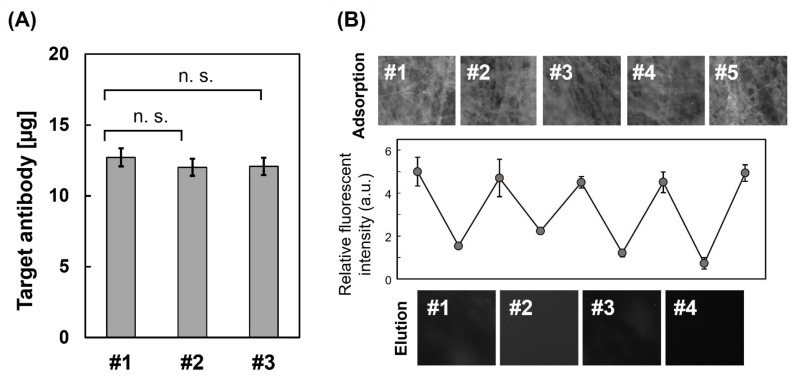
Successive repetitive adsorption-elution processes. (**A**) Amounts of antibody eluted (*n* = 3, n.s.: no significant difference), (**B**) Fluorescent microscopy of CA/PVA-PA NF, which were adsorbed with fluorescent-labeled IgG. The graph represents the quantitative evaluation of fluorescent intensity.

The adsorption-elution was repeated three times in succession to demonstrate the reusability as an affinity carrier. The antibody concentration at each adsorption was 300 µg/mL, as in the previous experiments, and the elution amount was measured at each elution. Thus, 30.7 µg of antibody was added each time, and the amounts eluted at the first, second, and third times were 12.7, 12.0, and 12.0 µg, respectively (Figure 8A). Assuming that the first elution amount was 100%, the second and third elution amounts were both 95%. Repetitive adsorption and elution steps within five times were also examined in the fluorescent microscopy to examine the stability of the fibrous structure (Figure 8B). It was found that the antibody capture ability was maintained and nanofiber morphology was sustained even after repeated adsorption and elution.

## 4. Discussion

In this study, it was shown that the core-shell fiber with a hydrogel layer on the surface captured the antibody with high efficiency. The dissociation constant of the antibody immobilized was higher than the previously reported ones. This would be because multiple IgG molecules bind to one PA [35], which leads to a decrease in apparent affinity. In this study, PA was immobilized not only at the fiber surface but in the 3D hydrogel shell-layer of PVA, which has the advantage that the diffusion length between PA and the antibody is lower than the 2D surface and the antibody could access PA from any direction readily [36]. To improve the accessibility of the antibody to PA, various site-specific immobilization methods, such as cysteine–maleimide reaction [37], peptide-tag conjugation [38], click-chemistry [39], and enzyme-mediated immobilization [40], would be applicable. In addition, the antibody used here was a wild type, but various recombinant PAs which are commercially available to improve their performance are also applicable. Core-shell nanofiber-based approach is perfectly compatible with those methods and this is the issue to be addressed in the future.

The amount of antibody loaded on an affinity carrier depends on the flow rate of the sample. The shapes of the affinity matrix, such as gel bead carriers and porous membrane carriers, affect the flow rate. Membrane carriers can also improve the flow-through performance with the high flow at a maximum rate of 10 mL/min. One commercially available membrane carrier (Sartobind Protein A 75 Membrane Adsorbers, Sartorius #85030-517-47) uses a membrane of 75 cm^2^ (2.1 mL) per device that adsorbs 0.08 mg/cm^2^ of antibody IgG [41]. This means that 6 mg of antibody can be captured per device and it takes approximately 200 s to adsorb 100 mg of antibody. Thus, the amount of antibody that can be adsorbed per unit reaction time is 29 mg.

The amount of antibody captured by the CA/PVA + PA NF fabricated in this study was 0.04 mg/cm^2^. If a device of the same size as a commercially available membrane carrier is constructed, the amount of antibody that can be captured per device is 3 mg. Assuming the same flow as conventional membrane chromatography (10 mL/min), it would be able to capture 14 mg/min of antibody. Moreover, owing to the nanometer-sized fibers, it has higher flow-through performance than commercially available porous membranes. It was previously reported that microporous poly(caprolactam) hollow NFs have a flow rate of 25 mL/min [42]. Therefore, the amount of antibody that can be adsorbed per unit time is 36 mg—a drastic improvement. The comparison with commercialized PA-immobilized substrate was summarized in Table 1. To increase the antibody-binding capacity by increasing the bed volume, NF membranes can be packed into columns and filters by layering sheets because they are thinner than porous membranes and beaded carriers.

## 5. Conclusions

In this study, we successfully electrospun protein-immobilized core-shell NFs in which the core of CA was covered with a shell of hydrophilic PVA hydrogel immobilized using PA. Conventional immobilization of proteins on the NF surface requires some steps after NF preparation, including activation of the surface, the addition of a cross-linking agent, and immobilization of proteins. Our method enabled one-step electrospinning, which would speed up the production. The bioactivity evaluation revealed that 12.9 mg/cm^3^ of antibody was adsorbed on the protein-immobilized fiber. The maximum adsorption site evaluated by the Langmuir model was 87.8 µg. This high activity originated from the hydrophilic environment of the PVA hydrogel, where the ligands were immobilized. The fiber sheet withstood triplicate use. Thus, our NF showed high potential as a material for membrane chromatography.

## Figures and Tables

**Table 1:** Comparison of fabricated NF with commercialized PA-immobilized substrate.

Carrier	Antibody Binding Capacity	Bed Volume	Max Flow Rate
Agarose gel beads ^1^	50 mg/mL(gel) = 100 mg/device	2mL (gel)	2 mL/min
CA porous membrane ^1^	0.08 mg/cm^2^ = 6 mg/device	2.1 mL (75 cm^2^)	10 mL/min
CA/PVA + PA NF (this study)	0.04 mg/cm^2^ = 3 mg/device	More than 2.1 mL (More than 75 cm^2^)	More than 10 mL/min

^1^ Manufacturers specifications from GE Healthcare for rProtein A Sepharose 4 Fast Flow and Sartorius AG for Sartobind Protein A 75 Membrane Adsorbers.

## Abbreviations

PA	protein A
CA	cellulose acetate
CDI	1,1’-carbonyldiimidazole
PVA	polyvinyl alcohol
NF	nanofiber
SEM	scanning electron microscopy
ATR-FTIR	attenuated total reflection Fourier-transform infrared spectrometry

## References

[1] Ayyar B.V., Arora S., Murphy C., O’Kennedy R. (2012). Affinity Chromatography as a tool for antibody purification. Methods.

[2] Avvakumova S., Colombo M., Tortora P., Prosperi D. (2014). Biotechnological approaches toward nanoparticle biofunctionalization. Trends. Biotechnol..

[3] Arora S., Saxena V., Ayyar B.V. (2017). Affinitiy chromatography: A versatile technique for antibody purification. Methods.

[4] Sjöquist J., Meloun B., Hjelm H. (1972). Protein A Isolated from Staphylococcus aureus after Digestion with Lysostaphin. Eur. J. Biochem..

[5] Kim D., Herr A.E. (2013). Protein immobilization techniques for microfluidic assays. Biomicrofluidics.

[6] Rusmini F., Zhong Z., Feijen J. (2007). Protein immobilization strategies for protein biochips. Biomacromolecules.

[7] Meldal M., Schoffelen S. (2016). Recent advances in covalent, site-specific protein immobilization. F1000Research.

[8] Smith S., Goodge K., Delaney M., Struzyk A., Tansey N., Frey M. (2020). A Comprehensive Review of the Covalent Immobilization of Biomolecules onto Electrospun Nanofibers. Nanomaterials.

[9] Sisson T.H., Castor C.W. (1990). An improved method for immobilizing IgG antibodies on protein A-agarose. J. Immunol. Methods.

[10] Yang L., Chen P. (2001). Chitosan/coarse filter paper composite membrane for fast purification of IgG from human serum. J. Membr. Sci..

[11] Denizli A., Arica Y. (2000). Protein A-immobilized microporous polyhydroxyethylmethacrylate affinity membranes for selective sorption of human-immunoglobulin-G from human plasma. J. Biomater. Sci. Polym. Ed..

[12] Ingavle G.C., Baillie L.W.J., Zheng Y., Lis E.K., Savina I.N., Howell C.A., Mikhalovsky S.V., Sandeman S.R. (2015). Affinity binding of antibodies to supermacroporous cryogel adsorbents with immobilized protein A for removal of anthrax toxin protective antigen. Biomaterials.

[13] Castilho L., Anspach F., Deckwer W. (2002). Comparison of affinity membranes for the purification of immunoglobulins. J. Membr. Sci..

[14] Zhang X., Wang Y., Zhong T., Feng X. (2019). Optimal Spacer Arm Microenvironment for the Immobilization of Recombinant Protein A on Heterofunctional Amino-Epoxy Agarose Supports. Process Biochem..

[15] Wang Z.G., Wan L.S., Liu Z.M., Huang X.J., Xu Z.K. (2009). Enzyme immobilization on electrospun polymer nanofibers: An overview. J. Mol. Catal. B Enzym..

[16] Ma Z., Kotaki M., Ramakrishna S. (2005). Electospun cellulose nanofiber as affinity membrane. J. Membr. Sci..

[17] Ahmed F.E., Lalia B.S., Hashaikeh R. (2015). A review on electrospinning for membrane fabrication: Challenges and applications. Desalination.

[18] Fujita S., Shimizu H., Suye S. (2012). Control of Differentiation of Human Mesenchymal Stem Cells by Altering the Geometry of Nanofibers. J. Nanotechnol..

[19] Wakuda Y., Nishimoto S., Suye S., Fujita S. (2018). Native collagen hydrogel nanofibres with anisotropic structure using core-shell electrospinning. Sci. Rep..

[20] Fujita S., Wakuda Y., Matsumura M., Suye S. (2019). Geometrically customizable alginate hydrogel nanofibers for cell culture platforms. J. Mater. Chem. B.

[21] Huang W.Y., Hibino T., Suye S., Fujita S. (2021). Electrospun collagen core/poly-l-lactic acid shell nanofibers for prolonged release of hydrophilic drug. RSC Adv..

[22] Barrett D.A., Hartshorne M.S., Hussain M.A., Shaw P.N., Davies M.C. (2001). Resistance to Nonspecific Protein Adsorption by Poly(vinyl alcohol) Thin Films Adsorbed to a Poly(styrene) Support Matrix Studied Using Surface Plasmon Resonance. Anal. Chem..

[23] Castilho L.R., Deckwer W.-D., Anspach F.B. (2000). Influence of matrix activation and polymer coating on the purification of human IgG with protein A affinity membranes. J. Membr. Sci..

[24] Wang F., Zhang H., Yu B., Wang S., Shen Y., Cong H. (2020). Review of the research on anti-protein fouling coatings materials. Prog. Org. Coat..

[25] de Jongh H.H., Goormaghtigh E., Ruysschaert J.M. (1996). The different molar absorptivities of the secondary structure types in the amide I region: An attenuated total reflection infrared study on globular proteins. Anal. Biochem..

[26] Beattie J.W., Rowland-Jones R.C., Farys M., Tran R., Kazarian S.G., Byrne B. (2021). Insight into purification of monoclonal antibodies in industrial columns via studies of Protein A binding capacity by in situ ATR-FTIR spectroscopy. Analyst.

[27] Movasaghi Z., Rehman S., Rehman I. (2008). Fourier Transform Infrared (FTIR) Spectroscopy of Biological Tissues. Appl. Spectrosc. Rev..

[28] Song J., Birbach N.L., Hinestroza J.P. (2012). Deposition of silver nanoparticles on cellulosic fibers via stabilization of carboxymethyl groups. Cellulose.

[29] Matharu Z., Bandodkar A.J., Sumana G., Solanki P.R., Ekanayake E.M.I.M., Kaneto K., Gupta V., Malhotra B.D. (2009). Low Density Lipoprotein Detection Based on Antibody Immobilized Self-Assembled Monolayer: Investigations of Kinetic and Thermodynamic Properties. J. Phys. Chem. B.

[30] Bayramoglu G., Ozalp V.C., Arica M.Y. (2013). Adsorption and separation of immunoglobulins by novel affinity core-shell beads decorated with Protein L and l-histidine. J. Chromatogr. B Anal. Technol. Biomed. Life Sci..

[31] Adamson A.W. (1990). Physical Chemistry of Surfaces.

[32] Nakanishi K., Tomita M., Masuda Y., Kato K. (2015). Gold nanoparticle–mesoporous silica sheet composites with enhanced antibody adsorption capacity. New J. Chem..

[33] Fahrner R., Iyer H., Blank G. (1999). The optimal flow rate and column length for maximum production rate of protein A affinity chromatography. Bioprocess Eng..

[34] Yang L., Biswas M.E., Chen P. (2003). Study of binding between Protein A and Immunoglobulin G Using a Surface Tension Probe. Biophys. J..

[35] Jansson B., Uhlén M., Nygren P.A. (1998). All individual domains of staphylococcal protein A show Fab binding. FEMS Immunol. Med. Microbiol..

[36] Salva M.L., Rocca M., Niemeyer C.M., Delamarche E. (2021). Methods for immobilizing receptors in microfluidic devices: A review. Micro Nano Eng..

[37] Zhang X., Duan Y., Zeng X. (2017). Improved Performance of Recombinant Protein A Immobilized on Agarose Beads by Site-Specific Conjugation. ACS Omega.

[38] Kaveh-Baghbaderani Y., Allgayer R., Schwaminger S.P., Fraga-García P., Berensmeier S. (2021). Magnetic Separation of Antibodies with High Binding Capacity by Site-Directed Immobilization of Protein A-Domains to Bare Iron Oxide Nanoparticles. ACS Appl. Nano Mater..

[39] Putti M., de Jong S.M.J., Stassen O.M.J.A., Sahlgren C.M., Dankers P.Y.W. (2019). A Supramolecular Platform for the Introduction of Fc-Fusion Bioactive Proteins on Biomaterial Surfaces. ACS Appl. Polym. Mater..

[40] Qafari S.M., Ahmadian G., Mohammadi M. (2017). One-step purification and oriented attachment of protein A on silica and graphene oxide nanoparticles using sortase-mediated immobilization. RSC Adv..

[41] Ma Z., Lan Z., Matsuura T., Ramakrishna S. (2009). Electrospun polyethersulfone affinity membrane: Membrane preparation and performance evaluation. J. Chromatogr. B.

[42] Klein E., Yeager D., Seshadri R., Baurmeister U. (1997). Affinity adsorption devices prepared from microporous poly(amide) hollow fibers and sheet membranes. J. Membr. Sci..

